# The Role of Cardiac Computed Tomography Angiography in Risk Stratification for Coronary Artery Disease

**DOI:** 10.1016/j.jscai.2024.102230

**Published:** 2024-11-20

**Authors:** Sophie E. van Rosendael, Arthur Shiyovich, Rhanderson N. Cardoso, Camila Veronica Souza Freire, Alexander R. van Rosendael, Fay Y. Lin, Gina Larocca, Solomon W. Bienstock, Ron Blankstein, Leslee J. Shaw

**Affiliations:** a Icahn School of Medicine at Mount Sinai, Mount Sinai Heart, Zena and Michael A. Wiener Cardiovascular Institute, and Marie-Josée and Henry R. Kravis Center for Cardiovascular Health, New York, New York; b Division of Cardiovascular Medicine, Department of Medicine, Brigham and Women’s Hospital, Harvard Medical School, Boston, Massachusetts; c Department of Cardiology, Leiden University Medical Center, Leiden, the Netherlands; d Department of Radiology, Brigham and Women’s Hospital, Harvard Medical School, Boston, Massachusetts

**Keywords:** coronary artery disease, coronary computed tomography angiography, major adverse cardiac event

## Abstract

Coronary computed tomography angiography (CCTA) allows the assessment of the presence and severity of obstructive and nonobstructive atherosclerotic coronary artery disease. With software developments incorporating artificial intelligence-based automated image analysis along with improved spatial resolution of CT scanners, volumetric measurements of atherosclerotic plaque, detection of high-risk plaque features, and delineation of pericoronary adipose tissue density can now be readily and accurately evaluated for a given at-risk patient. Many of these expanded diagnostic measures have been shown to be prognostically useful for prediction of major adverse cardiac events. The incremental value of plaque quantification over diameter stenosis has yet to be thoroughly discovered in current studies. Furthermore, the physiological significance of lesions can also be assessed with CT-derived fractional flow reserve, myocardial CT perfusion, and more recently shear stress, potentially leading to selective invasive coronary angiography and revascularization. Along with these technological advancements, there has been additional high-quality evidence for CCTA including large randomized clinical trials supporting high-level recommendations from many international clinical practice guidelines. Current trials largely compare a CCTA vs functional testing strategy, yet there is minimal evidence on CCTA plaque-guided therapeutic trials to measure regression of atherosclerosis and prevention of major coronary artery disease events. In this review, we summarize current evidence on comprehensive risk assessment with CCTA and future directions.

## Introduction

With 17.8 million deaths per year, coronary artery disease (CAD) is the most common cardiac pathology and is currently the third leading cause of death in the world.^1^ Coronary atherosclerosis, commences with activation of the endothelium before a cascade of events, namely lipid accumulation, fibrous elements and microcalcification, triggers activation of inflammatory pathways and ultimately narrowing of the arterial luminal diameter.^2^ These processes result in an atheromatous plaque, potentially causing anginal symptoms and complications. Various tests are currently available for the diagnostic evaluation of CAD, either direct detection of plaque or the functional consequence of myocardial ischemia. Ischemia can be assessed with noninvasive imaging tests such as exercise electrocardiography, myocardial perfusion imaging, or radionuclide scintigraphy. Coronary computed tomography angiography (CCTA) directly visualizes the plaque, independently of ischemia, and therefore may identify atherosclerotic risk at an earlier stage. Prior studies have shown a high sensitivity and good specificity of CCTA for the detection of CAD and also established the prognostic importance of anatomically mild, nonobstructive, plaques.^3^^,^^4^ Identification of which patients are at increased risk of major adverse cardiovascular events (MACE) is a topic of ongoing research, especially as the different therapies available to treat CAD continue to expand. With developments in CT scanner technology and analytic tools, atherosclerotic plaques can be accurately quantified. Common features assessed with CCTA are plaque extent and diameter stenosis, but quantification techniques now also enable the assessment of plaque volume, composition, and high-risk plaque (HRP) features.^5^, ^6^, ^7^ In addition, hemodynamic significance of coronary lesions can be measured noninvasively with CT-derived fractional flow reserve (FFR_CT_).^8^ Lastly, CCTA enables the evaluation of the pericoronary adipose tissue (PCAT), a biomarker of coronary inflammation, a driver of plaque progression, vulnerability, and rupture, and predictor of MACE. This review will provide an overview and a discussion of how CCTA aids in risk stratification of patients at risk for and with known CAD.

## Randomized trials with CCTA

There are now several randomized trials available, with most comparing CCTA to standard diagnostic testing. The trials range across a variety of patient populations from those presenting to the emergency department (ED) with acute onset of chest pain symptoms to those presenting for an outpatient evaluation of suspected CAD with de novo symptom evaluation.^9^ In total, there are 11 ED trials with most enrolling lower-risk patients with the results demonstrating that CCTA provided a rapid and accurate diagnosis of CAD which facilitated prompt discharge of patients with normal coronary arteries or minimal CAD but did not provide an improvement in longer-term outcomes when compared to standard testing approaches. Importantly, a general message from these trials was that there were no safety issues in promptly discharging patients including no adverse risk of death, myocardial infarction (MI), repeat ED visit, or ACS over ∼1 to 6 months when compared to standard testing, including stress testing.^9^ More recently, there are 2 trials that focus on a higher risk patient in the ED including those with acute MI, abnormal ECG, or elevated troponin.^10^^,^^11^ Among the higher-risk patients in these 2 latter trials, there were no differences in near-term outcomes. For example, in the report by Gray et al,^11^ use of an early CCTA (at ∼4.2 hours) did not yield improvement in 1-year death or MI (hazard ratio [HR], 0.91; *P* = .65).

In the stable chest pain setting of patients with suspected CAD, there are 9 published randomized trials enrolling more than 22,000 patients with comparisons largely to stress testing to compare near-term (2-3 year) effectiveness. None of the trials established an improvement in MACE over 2 to 3 years of follow-up. However, over a longer duration of follow-up (ie, 5 years), the Scottish Computed Tomography of the Heart (SCOT-HEART) trial demonstrated benefits in patients in whom CCTA was added to routine testing, with lower rates of cardiovascular death or nonfatal MI at 5 years.^12^ In addition, there are 2 trials that have compared an initial CCTA strategy to an invasive coronary angiography (ICA) strategy among patients with stable chest pain and intermediate pretest risk of CAD who qualify for elective ICA.^13^^,^^14^ In both studies, there were no differences in MACE between the CCTA and ICA strategies. For example, in the Diagnostic Imaging Strategies for Patients with Stable Chest Pain and Intermediate Risk of Coronary Artery Disease trial (n = 3561), the 1-year MACE rates were similar between CCTA and ICA strategies, with an HR of 0.70 (*P* = .10). A key message from both trials was that a lower rate of procedural complications occurred in the CCTA as compared to ICA arm of the trials.^13^

## Risk stratification among symptomatic patients

Coronary computed tomography angiography enables anatomic visualization of atherosclerosis in the whole coronary tree, among arterial diameters >2 mm. The presence, location, and extent of atherosclerotic plaque can be assessed, as well as its absence, the latter of which portends excellent prognosis with a low rate of MACE.^15^^,^^16^ Nielsen et al^15^ demonstrated a 1.5% event rate of the composite endpoint of death, MI, and coronary revascularization at 3.5 years of follow-up for patients with new-onset symptoms suggestive of CAD and a normal CCTA. Patients at an early stage of atherosclerosis with nonobstructive CAD (diameter stenosis <50%) can be identified by CCTA as well. Nonobstructive CAD may not correlate with cardiac symptoms or positive stress test findings but is associated with worsening prognosis as compared to patients with normal coronaries.^17^^,^^18^ The annual event rate for MACE for individuals with nonobstructive CAD is approximately 1.6%, whereas it was 0.2% for patients with no CAD, presenting an 8-fold higher rate among patients with nonobstructive disease.^19^

Atherosclerotic plaque burden provides value in cardiovascular risk prediction independently of how plaque burden is assessed. Increasing vessel involvement by plaque relates to MACE in a stepwise fashion.^20^, ^21^, ^22^ Other commonly used scores are the segment involvement score, the segment stenosis score, and the Coronary Artery Disease-Reporting and Data System (CAD-RADS) score.^3^^,^^23^^,^^24^ The segment involvement score, calculated by counting the number of coronary segments with atherosclerosis, is linearly associated with mortality.^4^^,^^25^, ^26^, ^27^, ^28^ Specifically, patients with chest symptoms and a score >5 had a mortality rate of 8.4% whereas patients with a score ≤5 had a mortality rate of 2.5% (*P* = .05).^3^ The segment stenosis score grades each segment based on the degree of stenosis (no plaque, mild, moderate, severe) which subsequently is summed leading to a score between 0 and 48, and has shown prognostic significance for MACE as well.^28^ Min et al^3^ showed an absolute difference in mortality rate of 5% between patients with a score >5 and ≤5 (6.6% vs 1.6%, *P* = .05).

The CAD-RADS scoring system is based on standardized categories of the most stenotic lesion on a scan, and graded as 0% (CAD-RADS 0), 1% to 24% (CAD-RADS 1), 25% to 49% (CAD-RADS 2), 50% to 69% (CAD-RADS 3), 70% to 99% (CAD-RADS 4) and 100% (CAD-RADS 5).^29^ CAD-RADS 4 has 2 subgroups, 4A including 1 or 2 vessels with 70% to 99% stenosis and 4B including 3-vessel obstructive (≥70%) disease or ≥50% diameter stenosis in the LM. Recently, CAD-RADS 2.0 was published and added a grading scale for the estimation of plaque burden (ranging from P1 to P4) and the possible assessment of ischemia by FFR_CT_ or CT perfusion.^24^ The CAD-RADS classification has demonstrated accurate predictive capability for MACE, including unstable angina, MI, or death. Better predictive performance has been shown for the CAD-RADS when compared to traditional cardiovascular risk factors, and the coronary artery calcium score (CACS).^30^, ^31^, ^32^

## Quantitative measurement of atherosclerotic plaque

The major advantage of quantification of atherosclerosis compared with visual estimation of plaque is a greater accuracy and reproducibility in measurement that may, in turn, improve prognostication. In some cases, quantitative plaque assessment has been shown to provide additional value beyond traditional CCTA assessment in risk stratification and has been validated against histology, IVUS, and OCT with good concordance.^33^, ^34^, ^35^, ^36^, ^37^, ^38^, ^39^, ^40^

Compositional plaque analyses are commonly based on Hounsfield units (HU) and categorized into 4 groups with slightly different HU ranges depending on the interpretive platform.^37^^,^^41^^,^^42^ The latest Society of Cardiovascular Computed Tomography expert consensus document describes: low-attenuation plaque for HU densities from −30 to 30 HU; fibrofatty plaque for HU values from 31 to 130; fibrous plaque for values ranging from 131 to 350 HU and dense calcium is defined as all voxels with HU >350.^19^ These values may differ when software packages use adaptive thresholding for instance adjusted for contrast attenuation in the coronary lumen. The association between quantitative plaque assessment and event prediction has been a topic of interest in research.^43^ Low-attenuation plaque, which is thought to correlate with a lipid-rich necrotic core of high-risk atheroma, has been shown to be an important marker of plaque instability and independent cardiovascular risk predictor.^5^^,^^7^^,^^44^^,^^45^

Williams et al^5^ demonstrated in a substudy of the SCOT-HEART trial that patients with a low-attenuation plaque burden >4% were 5 times more likely to suffer a subsequent fatal or nonfatal MI (HR, 4.65; 95% CI, 2.06-10.5; *P* < .001), independent of diameter stenosis, CACS, and overall plaque burden. Analyses of patients with nonobstructive lesions and >4% low-attenuation plaque burden showed an even higher HR for subsequent MI (HR, 6.61; 95% CI, 1.91 to 22.82; *P* = .003). De Knegt et al^46^ conducted a quantitative plaque analysis in a cohort comprising 274 asymptomatic individuals, 254 patients with acute chest pain without ACS, and 328 patients with ACS and found a significant increase in plaque volumes across the clinical risk profiles (148 mm^3^, 257 mm^3^, 407 mm^3^, respectively, *P* < .001). Furthermore, plaque morphology volumes differed between the 3 cohorts as well, with an increased proportion of fibrofatty and necrotic core and a decrease of dense calcium (fibrofatty: 50%, 61%, 57%; necrotic core: 17%, 17%, 20%; and dense calcium: 33%, 23%, 23%; respectively).

On the other hand, “brighter” calcified plaques on CCTA, the dense calcified plaque, have been associated with a reduced risk of events.^47^^,^^48^ Criqui et al^47^ demonstrated that overall calcium density score obtained by noncontrast CT was inversely related to coronary heart and cardiovascular disease risk after adjustment for overall calcium volume*.* Furthermore, a secondary analysis from the Incident Coronary Syndromes Identified by Computed Tomography study, showed that so-called “1K plaque,” dense calcified plaque with voxels above 1000 HU, was associated with a lower risk for future ACS.^48^

Dundas et al^49^ derived cutoffs for total plaque burden and every subtype using the Assessing Diagnostic Value of Non-invasive FFR-CT in Coronary Care registry and compared these with outcomes. They found that the total PAV above the cutoff was associated with both MACE/late revascularization (total PAV >24.4%; HR, 2.05; *P* < .001) and cardiovascular death/MI (total PAV >37.2%; HR, 4.53; *P* < .001). Elevated levels of calcified, noncalcified, and low-attenuation PAV were associated with all adverse outcomes; however, after stratification by median plaque volumes, this did not remain significant for cardiovascular death/MI.

**Figure 1:**
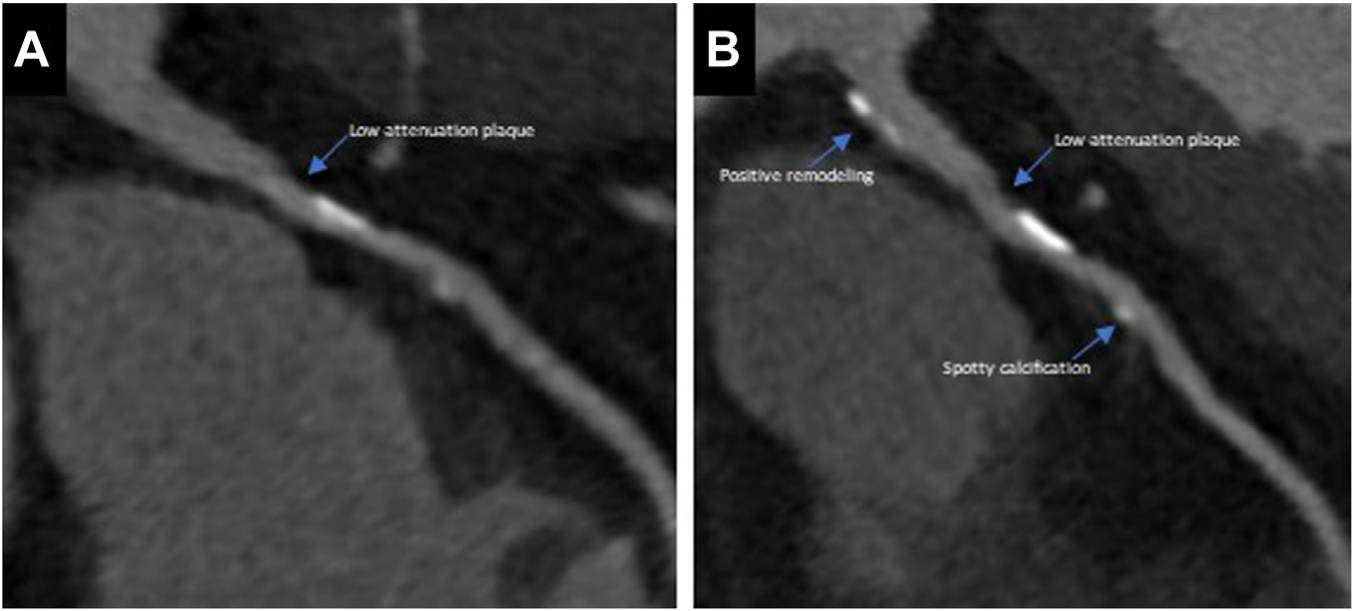
**Progression of coronary artery disease and high-risk plaque features on coronary computed tomography angiography (CCTA).** An example of a patient with plaque progression and an increase in high-risk plaque features shown on serial CCTA with (A) baseline and (B) follow-up CCTA of the proximal left anterior descending artery.

Besides plaque phenotypes, identification of HRP features has been used to identify high-risk lesions and provide incremental information in relation to the overall plaque burden. HRP features on CCTA include the napkin ring sign, low attenuation plaque (<30 HU), spotty calcification, and positive remodeling, and is defined by the CAD-RADS classification as a coronary lesion with at least 2 of these features (Table 1 and Figure 1).^24^ The qualitative assessment of HRP and findings on OCT and IVUS have shown good correlation in several studies.^50^, ^51^, ^52^ Kinoshita et al^53^ saw associations of all 4 HRP features with features of vulnerability on OCT, like thin-cap fibroatheroma. Feuchtner et al^7^ evaluated the prognostic value of HRP features and demonstrated that after adjustment for risk factors, diameter stenosis and plaque phenotype, low-attenuation plaque, and napkin-ring sign were the most powerful MACE predictors (HR, 4.50 and HR, 7.0; *P* < .001, respectively). Spotty calcification was less powerful (HR, 2.6; *P* < .001), and positive remodeling showed no significance before adjustment (HR, 1.69; *P* = .34).

**Table 1:** Definitions of high-risk plaque features.

High-risk plaque features	
Low-attenuation plaque	Presence of a central focal area with low CT attenuation, usually at least 1 voxel with HU below 30, within the plaque. Thresholds of HU below 60 and 90 are used as well.
Positive remodeling	The outer vessel diameter is at least 10% greater than the average diameter of the normal adjoining segments.
Napkin ring sign	Presence of circumferential necrotic core, a central area of low HU, that abuts the lumen, with a ring of high attenuation, not above 130 HU, surrounding this low attenuation area.
Spotty calcification	Focal calcification smaller than 3 mm diameter in any direction. Another description is the calcium burden length, in the longitudinal direction of the vessel, smaller than 1.5 times the vessel diameter, and width below two-thirds of the vessel diameter.

HU, Hounsfield units.

A substudy of the Prospective Multicenter Imaging Study for Evaluation of Chest Pain trial, evaluated the predictive value of HRP, defined as lesions with ≥1 HRP feature present.^6^ Lesions defined as HRP were, after adjustment for the atherosclerotic cardiovascular disease risk score and significant stenosis, predictive for MACE (HR, 1.72; 95% CI, 1.13-2.62). In patients with nonobstructive disease and HRP, the HR even doubled when compared to patients without HRP (HR, 4.31; 95% CI, 2.25-8.26 vs HR, 2.64; 95% CI, 1.49-4.69; respectively). In the Incident Coronary Syndromes Identified by Computed Tomography study, independent from diameter stenosis, volumes of low-attenuation and fibrofatty plaque were associated with increased risk for ACS, and HRP features offered the greatest prognostic utility to pinpoint patients who will experience future ACS.^41^

## Fractional flow reserve with CCTA

Coronary computed tomography angiography is a noninvasive imaging modality that offers the opportunity to integrate a functional measurement in addition to the prior discussed anatomical and quantitative coronary description, by the use of FFR_CT_. Considering nearly 50% of high-grade stenoses do not cause ischemia, and on the contrary, a proportion of nonobstructive lesions manifest ischemia by invasive fractional flow reserve (iFFR), which implies that the correlation between physiological and angiographic stenosis severity may not be optimal.^54^ However, prior research reveals the relevance of identifying lesions that cause ischemia, which helps for management of symptomatic patients and potentially provides incremental anatomical features for prognosis.^55^^,^^56^

Using computational fluid dynamics principles and the 3-dimensional anatomical and physiological models retrospectively derived by coronary CT images, a 3-dimensional pressure map is made and provides a physiological assessment of coronary atherosclerosis of all coronary segments simultaneously (Central Illustration).^8^

**Central illustration:**
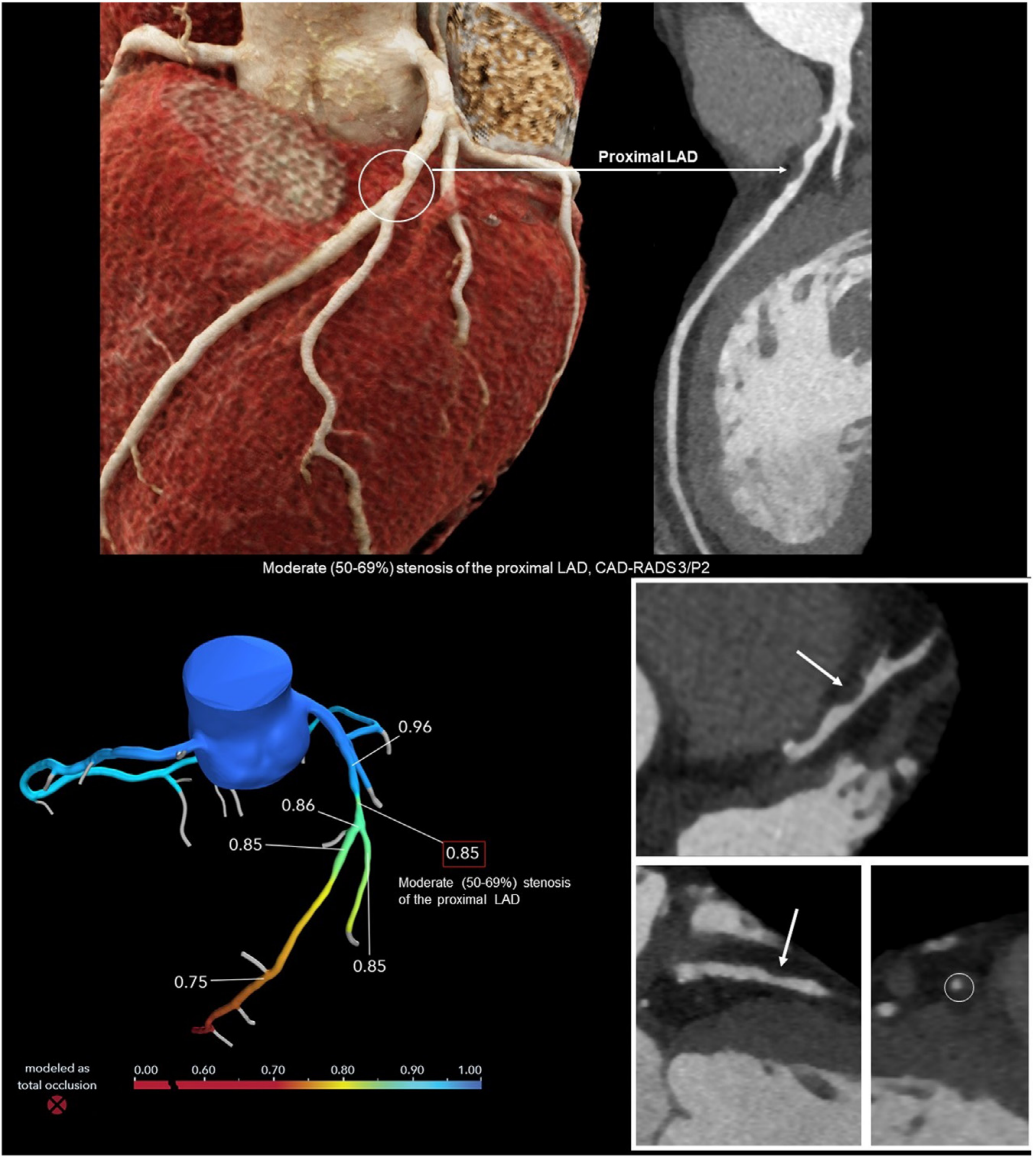
CT-derived fractional flow reserve (FFR_CT_) of the left anterior descending artery with moderate stenosis of the proximal LAD with a negative FFR_CT_.

A prospective subanalysis of the Analysis of Coronary Blood Flow Using Coronary CT Angiography: Next Steps trial showed an excellent correlation between FFR_CT_ and iFFR (Pearson's correlation coefficient 0.82; *P* < .001).^57^ In addition, a positive FFR_CT_ (≤0.8) in patients with stable CAD was superior to anatomically significant stenosis (diameter stenosis >50%) and was a significant predictor of MACE, driven by planned and unplanned revascularization (HR, 5.5; 95% CI, 1.6-19.0; *P* = .006). Madsen et al^58^ studied 900 individuals with new-onset angina who underwent FFR_CT_ if there was at least 1 lesion with diameter stenosis greater than 30%. The FFR_CT_ was obtained in the most stenotic lesion of each coronary artery and successfully stratified risk between patients with and without normal FFR (≤0.80). The rate of the composite end point of nonfatal MI and all-cause death at 3 years in patients with normal FFR was 2.1% and in patients with an abnormal FFR was 6.6% (relative risk, 3.1; 95% CI: 1.6, 6.3; *P* < .001). This increased risk in patients with an abnormal FFR persisted after adjustment for degree of stenosis and CAC score (relative risk, 2.5; 95% CI, 1.2-5.2; *P* = .01).

A head-to-head comparison of noninvasive coronary artery imaging, The PACIFIC trial, showed that positron emission tomography (PET) was the most accurate diagnostic test in diagnosis of myocardial ischemia compared to CCTA or single photon emission computed tomography using iFFR as reference standard.^59^ A post hoc analysis added FFR_CT_ into this comparison and showed a very good diagnostic performance for identification of ischemia on a per-vessel level as FFR_CT_ outperformed all 3 other modalities (area under the receiver-operating characteristic curve [AUC] for FFR_CT_, 0.94; AUC for CCTA, 0.83; *P* < .01; AUC for SPECT, 0.70; *P* < .01; AUC for PET: 0.87; *P* < .01).^60^ However, per-patient analyses showed better performance of PET (AUC FFR_CT_, 0.91 and AUC PET, 0.92; *P* = .56) in identifying ischemia, and in intention-to-diagnose analyses, PET performed better than FFR_CT_ on both per-patient and per-vessel levels (AUC PET, 0.86 vs AUC FFR_CT_, 0.83; *P* = .157; and AUC PET, 0.90 vs AUC FFR_CT_, 0.79; *P* = .005, respectively).

The 1-year outcomes from the Assessing Diagnostic Value of Non-invasive FFR-CT in Coronary Care registry including 4288 patients, revealed that patients with an FFR_CT_ ≤0.80 were 4 times more likely to experience MI or cardiovascular death than patients with FFR_CT_ >0.80 (25 [0.80%] vs 3 [0.20%]; relative risk, 4.22; 95% CI, 1.28-13.95; *P* = 0.01). Additionally, revascularization occurred less in the FFR_CT_ >0.80 group (1208 [38.40%] vs 89 [5.60%]; relative risk, 6.87; 95% CI, 5.59-8.45; *P* < .001) and was uncommon 90 days after the test (4.4% within 90 days, 1.20% between days 91 and 365).^61^

In addition, noninvasive hemodynamic analysis on CCTA, including assessment of FFR_CT,_ ΔFFR_CT,_ wall shear stress, and axial plaque stress, has been demonstrated to enhance the identification of HRP in patients at risk for cardiac events.^62^ The investigators of the EMERALD study^62^ found that the integration of noninvasive, lesion-specific physiology assessment improved the identification of culprit lesions for subsequent ACS. The EMERALD II trial investigated the performance of AI-enabled CCTA-derived quantitative plaque and hemodynamic analysis for ACS prediction and found that the best AI-enabled quantitative plaque features were ΔFFR_CT_, plaque burden, total plaque volume, low attenuation plaque volume, and averaged percent total myocardial blood flow. The authors concluded that AI-enabled quantitative plaque and hemodynamic analysis has the potential to enhance the prediction of lesion-specific ACS risk when added to conventional CCTA analysis, and integration of these algorithms in clinical practice can improve risk stratification to prevent ACS and optimize CAD treatment strategy.^63^

The effect of FFR_CT_ on clinical costs was studied in the FORECAST trial, showing that routine management vs the use of CCTA with selective FFR_CT_ decreased the use of ICA but did not reduce revascularization and costs.^64^ In addition, both groups had similar clinical outcomes including angina, quality of life, MACE, and cerebrovascular events after 9 months. The PRECISE trial demonstrated in 2103 patients with stable chest pain that the specific use of CCTA with FFR_CT_ according to the patients’ risk status, led to less frequent ICA and increased diagnostic yield for obstructive disease, without an increase in nonfatal MI or death at 1 year compared to usual testing and care.^65^

Studies have been performed to assess the utility of FFR_CT_ to guide coronary procedures in the catheterization laboratory. The so-called FFR_CT_ planner is a novel tool allowing virtual stenting of stenosis and prediction of FFR post percutaneous coronary intervention (PCI), based on changes to patient-specific lumen geometry.^66^ Post-PCI FFR is a metric of the degree of functional revascularization, with higher post-PCI FFR values associated with a better prognosis.^67^^,^^68^ Sonck et al^66^ found that the FFR_CT_ planner accurately predicted post-PCI FFR with high accuracy using invasively measured post-PCI FFR as reference, demonstrating its potential in predicting physiological benefits of PCI. Furthermore, the FASTTRACK CABG trial,^69^ a prospective, multicenter study, assessed the safety and feasibility of planning and execution of bypass surgery in patients with complex CAD, based on CCTA combined with FFR_CT_. The investigators found that in 99% of the included patients with a high disease burden who were candidates for coronary artery bypass grafting, treatment planning using CCTA and FFR_CT_ was feasible, offering a safe alternative to ICA.

It is important to highlight that image quality is a critical component of FFR_CT_ analysis. In the PACIFIC study, 83% of all arteries were deemed of sufficient quality for FFR_CT_ evaluation.^60^ This indicates that use of FFR_CT_ in clinical practice largely depends on good-quality scans arising from sufficient prescan medications and high-quality scanners. Mickley et al^70^ observed moderate diagnostic accuracy of FFR_CT_ to identify hemodynamically significant CAD in patients with an Agatston score above 399 using FFR/ICA as a reference. The sensitivity was 95% but the specificity was only 32%. When the FFR_CT_ values were colocalized to the approximate site of iFFR measurements, the specificity improved but remained low at 52%.

## PCAT

Pericoronary adipose tissue, a biomarker associated with vascular inflammation, is postulated to provide added risk information.^71^ Detection and quantification of vascular inflammation may enhance early risk assessment of patients. Vascular inflammation is a key factor in coronary atherosclerotic plaque formation, progression, and rupture and affects the differentiation, proliferation, and lipolysis of the adipocytes in the fatty tissue around the coronary arteries.^71^, ^72^, ^73^, ^74^, ^75^, ^76^ This leads to smaller adipocytes with lower intracellular lipid content which is correlated with higher HU, or attenuation values, on CCTA. The feasibility of PCAT attenuation obtained with CCTA and vascular inflammation detection has been shown.^71^^,^^77^^,^^78^ Significantly different PCAT attenuation values have been identified between coronaries with and without atherosclerotic plaque, within culprit and nonculprit lesions, and between flow-limiting and non–flow-limiting stenosis.^77^, ^78^, ^79^, ^80^ Lin et al^81^ observed significant differences in HU of PCAT of the proximal right coronary artery (RCA) in patients with an MI, stable CAD and no CAD (acute MI, –83.1 [–86.6 to –79.8] HU; stable CAD, –90.4 [–95.2 to –86.6] HU; no CAD, –93.7 [–98.2 to –87.9 HU], *P* < .001). The proximal RCA is characterized by the highest volume of surrounding adipose tissue and by the absence of confounding nonfatty structures such as side branches, myocardium, or coronary veins.^71^^,^^82^ However, prior studies analyzing differences between the 3 coronary arteries show significant differences in PCAT values.^79^^,^^83^^,^^84^ The Cardiovascular Risk Prediction using Computed Tomography study incorporated PCAT attenuation values in a propriety algorithm (CaRiHEART, Caristo Diagnostics) to calculate the fat attenuation index (FAI) and validated its prognostic value.^85^ A difference in the prognostic value of the 3 coronary arteries was observed, but when measured around the proximal RCA, the perivascular FAI improved cardiac risk prediction beyond current best practice assessment of coronary CTA. High perivascular FAI values ≥70.1 HU after adjustment were an indicator of increased cardiac mortality in both derivation and validation cohorts (HR, 9.04; 95% CI, 3.35-24.4; *P* < .001 and HR, 5.62; 95% CI, 2.90-10.88; *P* < .001; respectively).

In contrast, Chatterjee et al^86^ evaluated the predictive value of PCAT attenuation for MACE in high-risk patients referred for ICA but reported no predictive value of PCAT for events in any of the 3 coronary arteries during 5 years follow-up (RCA, 0.96; 95% CI, 0.75-1.22; *P* = .71, LAD, 1.31; 95% CI, 0.96-1.78; *P* = .09, and LCx, 0.98; 95% CI, 0.78-1.22; *P* = .84; respectively). Other study results are ambivalent as well, with a positive study by van Diemen et al^87^ demonstrating incremental value of PCAT values of the proximal RCA beyond clinical and quantitative plaque characteristics and ischemia, whereas no added prognostic value was found beyond the CAD-RADS by Wen et al^88^ in patients with acute chest pain. Independent of cardiovascular risk factors, elevated PCAT attenuation has been shown to be associated with the presence of noncalcified plaque^78^ and HRP.^89^ Tzolos et al^90^ reported in patients with stable chest pain a significant predictive effect of PCAT measured around the RCA for the risk of future MI. When RCA PCAT was added to low-attenuation plaque burden >4%, it led to improved prediction of future MI.

The association of PCAT and cardiovascular medical therapy is not well explored yet. One study evaluated the effect of statin therapy on PCAT and did not find significant differences in PCAT attenuation values at follow-up between 31 statin-taking patients and 70 statin-naïve patients (−1.61 ± 6.82 HU vs +1.39 ± 7.87 HU; *P* = .065).^78^

Currently, there is no consensus yet on the gold standard of how to measure PCAT, which leads to many different methods. Studies that include the proximal part of 1 coronary artery (mainly the RCA) or all 3 coronaries are more/most often published,^78^^,^^80^^,^^81^^,^^85^, ^86^, ^87^^,^^89^, ^90^, ^91^, ^92^, ^93^, ^94^, ^95^, ^96^, ^97^ along with lesion-specific PCAT analyses, ranging from assessment of the most stenotic lesion only, to culprit lesions, or the average of all lesions in 1 patient.^81^^,^^89^^,^^93^^,^^96^^,^^98^, ^99^, ^100^

While assessing PCAT, specific image acquisition and patient features might be considered confounding variables. Considering technical factors, significant differences in mean PCAT attenuation based on the CT scanner type used have been demonstrated in prior studies, and positive associations between PCAT and tube voltage, current, and pixel spacing have been discovered.^79^^,^^87^^,^^97^ Boussoussou et al^97^ determined in their study that the association between PCAT and noncalcified plaque did not hold after adjustment for these imaging characteristics and the patients’ heart rate. Adjustments for potential confounding factors vary widely within studies, and more research is needed to better understand which factors influence this parameter and how to adjust for them. Furthermore, the association of PCAT with atherogenesis and events, and the optimal way to implement this parameter clinically, are poorly understood and necessitate further research.

## Sex differences on CCTA identified atherosclerosis

Significant sex differences in pathophysiology of ischemic heart disease are identified in prior research.^101^ An approximate 10-year delay in onset of atherosclerosis is often reported in women when compared with men, and sex-specific atherosclerotic plaque profiles have been described. Women have less calcified and a high percent of nonobstructive CAD, with the potential to have more frequent coronary microvascular dysfunction as an etiology for symptoms.^102^, ^103^, ^104^, ^105^, ^106^, ^107^ In the setting of more extensive CAD, acute and long-term mortality risk is higher among women, especially those with 2-vessel or 3-vessel/left main disease.^16^ A post hoc analysis of the CONFIRM registry demonstrated a significantly increased risk of events in women compared with men when categorized in the same atherosclerotic burden group using a risk score.^105^ Women in the highest atherosclerotic burden group (score >20) had an HR of 6.71 (95% CI, 4.36-10.32) whereas men had a hazard score of 2.38 (95% CI, 1.73-3.29) (adjusted *P*-interaction = 0.003). Specifically, postmenopausal women (aged ≥55 years) with a high disease burden demonstrated a notable increase in risk relative to their male counterparts, with an HR of 6.11 (95% CI, 3.84-9.70) vs 2.25 (95% CI, 1.58-3.22) for men (adjusted *P*-interaction = 0.004). Despite lower cardiovascular risk in premenopausal women compared to age-matched men, the risk increases significantly after menopause.

Age- and sex-specific nomograms for quantitative atherosclerotic plaque on CCTA are derived from 11,808 patients, presenting age- and sex-stratified distributions of plaque subtypes assessed with artificial intelligence plaque analysis software.^108^ Women showed lower volumes of total plaque volume and all subcomponents than men. Both sexes showed an increase in plaque volumes with increasing age, with a shift from a higher proportion of noncalcified plaque to a higher proportion of calcified plaque.

A higher discriminatory value of atherosclerotic plaque to predict MACE is found in women.^104^^,^^109^ Among 1127 patients, the extent of nonobstructive CAD was found to be of prognostic value in women, but not in men.^18^ In addition, Xie et al^109^ found a significantly higher predictive value of nonobstructive CAD in the left main coronary artery leading to cardiac events in women than in men.^95^ Furthermore, Ferencik et al^6^ demonstrated that HRP was a stronger predictor of MACE in women compared to men, even after adjusting for stenosis severity (HR, 2.41; 95% CI, 1.25-4.64 vs HR, 1.40; 95% CI, 0.81-2.39, respectively).

Sex-specific plaque burden and its association to ischemia obtained with iFFR was studied by Han et al.^110^ They found lower stenosis severity, plaque volumes of total, calcified, noncalcified, and low-density plaque, and fewer adverse plaque characteristics, in women compared to men. In ischemic vessels, only low attenuation plaque was significantly lower in women (β: −0.183; *P* = 0.035), whereas the other plaque morphologies showed no difference by sex. All plaque subtype burdens were independently associated with abnormal FFR (≤0.80) in both sexes, without a significant interaction for the prediction of ischemia between sex and total plaque amount (interaction *P* = 0.108). Importantly, women have smaller coronary arteries and a lower myocardial mass, which leads to a higher coronary volume to myocardial mass ratio for the same degree of diameter stenosis compared to men.^111^ Women are therefore, despite a similar degree in diameter stenosis, less likely to have an FFR_CT_ ≤0.80 compared to men, potentially leading to sex-differences in referral for revascularization.

## Screening of asymptomatic individuals with CCTA

The majority of patients who develop MACE have no prior cardiac symptoms or manifestations of CAD,^112^ and more than 50% of acute coronary syndromes arise from previously documented nonobstructive lesions.^17^^,^^41^ It seems to be reasonable then that the detection of CAD in the asymptomatic individual is worthy of consideration. In general, identification of patients at risk is recommended using established cardiovascular risk scores with the addition of CACS.^113^ Certain scores have undergone updates in the past years to incorporate new factors like inflammatory conditions; however, their accuracy in estimating disease prevalence remains imperfect.^114^ This is particularly the case when applied to underrepresented populations in the literature, like women and diverse ethnic groups.^115^ A Cochrane systematic review and meta-analysis included 41 randomized controlled trials and showed that the use of cardiovascular risk scores compared to usual care, reduced total cholesterol, systolic blood pressure, and multivariable cardiovascular risk.^116^ The authors stated that the use of a cardiovascular risk score potentially reduced the adverse events rate, but the results were imprecise (1.9% vs 2.7%; RR, 0.72; 95% CI, 0.49-1.04; I^2^ = 0%; 4 trials, N = 4630, low-quality evidence). Preventive medication prescription increased in this study among patients identified as higher risk with no apparent evidence of harm.

The CACS visualizes calcified lesions, by which it provides a marker of atherosclerosis. It is quick and associated with minimal radiation exposure (∼1-2 mSv). Many studies have shown it improves prediction of MACE above and beyond clinical risk scores (MESA studies). However, in studies showing that noncalcified plaque occurs first, CACS would be considered a late marker of CAD risk. The SCAPIS trial included asymptomatic patients and demonstrated a prevalence of any atherosclerosis on CCTA of 42.1%.^117^ Moreover, among patients with a CACS of 0, atherosclerosis was present in 8.1% of diabetics, 6.0% in patients with a strong family history of MI, and 6.8% in current smokers.^117^ This highlights the prevalence of atherosclerosis as a silent disease. FACTOR-64, the only randomized controlled trial in asymptomatic patients to date, included 900 patients with diabetes mellitus who either underwent CCTA imaging or not, with a follow-up time of 4 years.^118^ The CCTA arm showed a 20% reduction in MACE, but did not reach statistical significance (HR, 0.8; 95% CI, 0.49-1.32; *P* = .38). Important in this study is that only diameter stenosis on CCTA was measured, which might have reduced the precision of prognostication.

However, CCTA has some important limitations. The radiation exposure for CCTA is higher compared to calcium scoring. Furthermore, the use of contrast and if necessary, the admission of β-blockers and nitroglycerin, can potentially cause side effects and/or adverse reactions.

The SCOT-HEART 2 trial has started enrolling 6000 asymptomatic individuals at risk of coronary heart disease to determine whether CCTA-based screening is associated with changes in medical therapy and rates of development of CAD, compared with standard of care, which includes the use of a Scottish risk score. Another study is the TRANSFORM trial, which aims to enroll 7500 asymptomatic patients with prediabetes, diabetes mellitus type 2, or metabolic syndrome who will all undergo CCTA. The aim is to prove that for the primary prevention of MACE, a personalized care strategy, including CAD plaque staging system reports, is better than usual care based on ASCVD risk factors.

## Serial CCTA

It has been demonstrated that early identification of CAD and timely implementation of preventative measures lower the risk of subsequent cardiovascular events.^12^ Plaques undergo morphological progression over time, and this can be measured through serial CCTA.^119^, ^120^, ^121^, ^122^, ^123^, ^124^, ^125^ Plaque progression has been associated with higher risk of long-term mortality,^126^ and the quantification of progression using CT imaging may enhance prognostication and help guide the utilization of preventive therapy. Motoyama et al^127^ demonstrated that plaque progression independently predicted ACS, with adverse events in 14.3% of 56 individuals in the plaque progression group and 0.3% of 367 patients in the group with no plaque progression (HR, 33.43; 95% CI, 4.13 to 78.03; *P* = .0006). The Progression of Atherosclerotic Plaque Determined by Computed Tomographic Angiography Imaging registry is a multinational registry, including individuals with serial CCTA at least 2 years apart with the objective of understanding the nature of atherosclerosis progression and the factors associated with it.^128^^,^^129^ They showed that atherosclerosis progression, independent from baseline plaque volume, has prognostic significance.^42^^,^^130^ Specifically, an annual increase of 1.0% percent atheroma volume was associated with a higher rate of events. Additionally, quantitative plaque assessment was more important in identifying patients at risk of rapid coronary plaque progression than clinical, laboratory, and qualitative measures.^131^ Pattern evaluation of plaque progression from nonobstructive to obstructive lesions according to the presence of HRP showed that baseline PAV and percent diameter stenosis were predictive (HR, 1.04; 95% CI, 1.02-1.07; and HR, 1.07; 95% CI, 1.04-1.10; respectively, all *P* < .05).^120^

**Figure 2:**
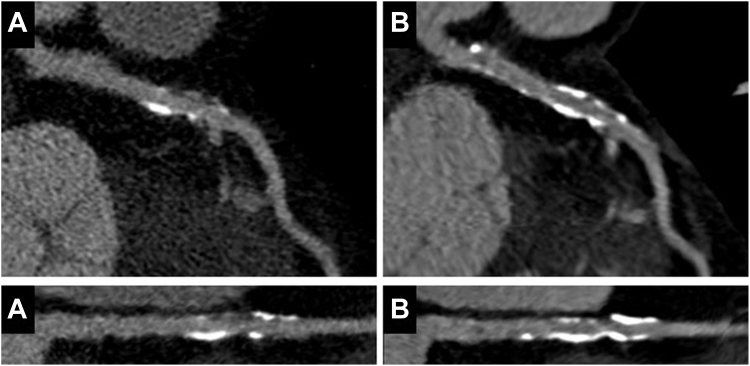
**Serial CCTA with initiation of statin therapy after baseline coronary computed tomography angiography.** Example of plaque on (A) baseline and (B) follow-up of the proximal left anterior descending artery in a patient with coronary artery disease. Statin therapy is started after the baseline coronary computed tomography angiography. The follow-up CT demonstrates progression of mainly calcified plaque.

Moreover, the effect of lipid-lowering therapies on plaque morphology and progression has been studied and evidence is available regarding the impact of statins on the progression of atherosclerosis. Statins have been shown to attenuate plaque progression with slower progression of noncalcified plaque and larger progression of calcified plaque.^132^^,^^133^ Figure 2 provides an example of a patient with serial CCTA and statin therapy initiation after baseline CT. Lee et al^129^ demonstrated similar results with slower progression of overall plaque volume in statin users compared to statin-naïve patients (annualized percent atheroma volume increase: 1.76 ± 2.40% vs 2.04 ± 2.37%, respectively; *P* = .002), with increased progression of calcified percent atheroma volume (1.27 ± 1.54% per year vs 0.98 ± 1.27% per year, respectively; *P* < .001), and lower progression of noncalcified percent atheroma volume (0.49 ± 2.39% per year vs 1.06 ± 2.42% per year) and incidence of HRP features (0.9% per year vs 1.6% per year, respectively; all *P* < .001).^98^ Patients at risk for plaque progression despite statin therapy, defined as an annual ≥1.0% increase in percent atheroma volume, were patients with high plaque burden and HRP at baseline.^134^ In a meta-analysis including 12 studies, intensive statin therapy demonstrated a reduction of total plaque volume by 20.87 mm^3^ or 3.6% compared to a 14.96 mm^3^ or 5.8% increase observed in control groups (*P* = .002).^135^ Additionally, statin therapy decreased noncalcified plaque volume by 7.62 mm^3^ and low-attenuation plaque volume by 5.84 mm^3^ and increased calcified plaque volume by 11.83 mm^3^.

The EVAPORATE trial evaluated whether icosapent ethyl, previously shown to reduce risk of ischemic events in patients with elevated triglyceride levels,^136^ given in addition to diet and statin therapy, would cause a larger change in plaque volume than in patients treated with statins and placebo.^137^ A regression of low-attenuation plaque volume at 18 months was seen in patients treated with icosapent ethyl, whereas the volume of low-attenuation plaque in the placebo group doubled (−17% vs +109%; *P* = .0061). In addition, a reduction of fibrous, fibrofatty, total noncalcified, and total plaque volume was seen in the icosapent ethyl group whereas there were increases of all plaque volumes in the placebo group (−20% vs +1%, *P* = .0028; −34% vs +32%, *P* = .0002; −19% vs +9%, *P* = .0005; −9% vs +11%, *P* = .0019; respectively). Alfaddagh et al^138^ found that eicosapentaenoic acid and docosahexaenoic acid added to statins in nondiabetic patients with mean LDL cholesterol <80 mg/dL, prevented coronary plaque progression of all plaque morphologies when an omega-3 index ≥4% was achieved.

Patients with an elevated lipoprotein(a) [Lp(a)] level were associated in a prior OCT study with more frequent thin-cap fibroatheroma.^139^ Kaiser et al^140^ studied the association of Lp(a) and plaque progression, showing that patients with high Lp(a) (≥ 70 mg/dL) had significantly accelerated progression of low-attenuation plaque compared with patients with low Lp(a) (26.2 ± 88.4 mm^3^ vs −0.7 ± 50.1 mm^3^; *P* = .020). No differences were found in total, noncalcified, and calcified plaque progression between the 2 groups.

Pontone et al^141^ established a risk score in order to predict disease development in patients with a low-to-intermediate chance of CAD. New occurrence of HRP and/or ≥50% diameter stenosis was classified as atherosclerosis progression and occurred more often in patients with a higher segment involvement score, HRP features like spotty calcification and low attenuation plaque, and diameter stenosis between 25% to 49% at baseline coronary CT. Independent predictors of plaque progression were included in the model: scan interval between CCTA scans (OR, 1.21; 95% CI, 1.02-1.42; *P* = .026), number of plaques with low-attenuation plaque (OR, 3.73; 95% CI, 1.46-9.52; *P* = .006), number of plaques with spotty calcification (OR, 4.59; 95% CI, 1.69-12.48; *P* = .003), number of bifurcation plaques (OR, 1.47; 95% CI, 1.17-1.84; *P* = .001), and stenosis severity of 25% to 49% (OR, 2.71; 95% CI, 1.62-4.50; *P* < .001). Based on the weight of regression coefficients, a score was obtained and was able to distinguish between low- and high-risk patients with similar C-statistics in the derivation and validation cohorts (0.732 [0.676-0.788] and 0.668 [0.583-0.752], respectively). To understand which patient would benefit most from repeat CCTA, validation of the risk score in larger cohorts is necessary.

The clinical use of serial CCTA in patients on guideline-directed management and therapy, with no change in clinical or functional status, received the class 3 recommendation in the 2023 American Heart Association (AHA)/American College of Cardiology (ACC) Multisociety Guideline for the Management of Patients with Chronic Coronary Disease.^142^ There is no clear recommendation in patients with changes in clinical or functional status, but we can anticipate that in certain subgroups with concerning quantitative plaque features on CCTA such as a high low-attenuation plaque burden or HRP features, repeat evaluation may be beneficial, based on its capability to assess plaque progression over time. In those patients, it could potentially be used to guide the intensity of medical therapy. In patients with rapid plaque progression (ie, an increase in plaque volume or development of HRP), intensified preventive care would be a part of the therapeutic strategy.

Of note, the rapid advancement in CT technology and the diverse types of scanners in the clinical setting pose significant issues for employing serial CCTA strategies. Multiple studies have used different CT scanners with different specifications at baseline and follow-up.^120^^,^^121^^,^^131^ Symons et al^143^ studied scanner variability in 40 individuals showing lower scan-rescan reproducibility in patients scanned with 2 different brand CT scanners. The scanner variability was approximately 18.4% (coefficient of variation) when the same scanner was used for both scans, whereas the scanner variability was ± 29.4% when different scanners were used. In addition, Takagi et al^144^ reviewed in 1236 patients from the Progression of Atherosclerotic Plaque Determined by Computed Tomographic Angiography Imaging registry, the impact of tube voltage on quantitative plaque quantification and composition on CCTA. Scanning with higher tube voltages (from 80 kV to 100 kV, and to 120 kV) resulted in decreased luminal HU and calcified plaque volume, and increased fibrofatty plaque and necrotic core volumes.

## Photon counting and ultrahigh spatial resolution CCTA

The diagnostic reliability of CCTA is closely linked to image quality. Factors influencing image quality include body habitus, heart rate as well as acquisition parameters, and scanner hardware.^145^ In addition, blooming artifacts, partial volume effects beam hardening from calcification, and artifacts related to motion affect the interpretability of the CCTA, especially in smaller vessels (<2 mm). However, ultrahigh resolution (UHR) CCTA with photon counting CT is a promising new type of scanner having excellent image quality and reduced blooming induced by calcium.^146^ Photon counting CT uses new, energy-resolving x-ray detectors to count and categorize incoming photons based on their energy and convert them into electrical signals.^147^ Measurement speed increases with this 1-step conversion which allows measurement of all x-ray photons individually within a single projection, with optimized geometric dose efficiency at very high spatial resolution.^148^ In addition, a UHR mode is available with a maximum in-plane resolution of 0.11mm.^149^ It leads to an absence of electric noise and enhances visualization of stent patency, smaller distal coronary segments, and coronary calcification.^150^

Latina et al^151^ assessed the diagnostic accuracy in 15 high-risk patients who underwent UHR-CT and ICA for suspected CAD. In this small sample, 7 patients were obese, 8 patients had at least 1 intraluminal stent and every patient had to have at least severe stenosis (>50% stenosis) at prior imaging, or a calcium score >400 or a stent. The studies with the UHR-CT were all interpretable and showed a high diagnosis accuracy with a high sensitivity (86%; 95% CI, 65%-97%) and specificity (88%; 95% CI, 77%-95%) in a per-vessel analysis vs ICA. Motoyama et al^152^ studied 79 patients with 102 calcified lesions and 79 stents, and showed, compared to conventional-resolution CT, improved median stenosis grading on UHR-CT, a significantly larger in-stent lumen, and significantly thinner stent struts. An additional 59 individuals also underwent ICA within 3 months of the UHR-CT, and per-patient analysis demonstrated high sensitivity (100%) and specificity (80%) for the detection of severe stenosis (≥70%), with a positive predictive value of 93.6% and a negative predictive value of 100%. Sensitivity and negative predictive value were similar in per-segment analysis (both 100%), the specificity was 95.8%, and a positive predictive value of 79.5% was found. Hagar et al^153^ assessed plaque severity in 68 high-risk patients using dual-source UHR photon counting CT. The individuals were considered for transcatheter aortic valve replacement and underwent ICA. The AUC for the detection of CAD with UHR photon-counting CT was 0.93 per patient, 0.94 per vessel, and 0.92 per segment. Per-patient analysis showed for the detection of CAD with ≥50% stenosis a high accuracy (88%), sensitivity (96%), and specificity (84%), and for detection of obstructive CAD (≥70% diameter stenosis) an accuracy of 83%, sensitivity of 100%, and specificity of 76%. Despite a high coronary artery calcium score (≥1000), a prior stent, or known history of CAD, the accuracy for identification of stenosis ≥50% remained high (83% in patients with Agatston score ≥1000 vs 91% in patients with a CACS <1000) but decreased in the identification of stenosis ≥70% in these patients (71% in patients with Agatston score ≥1000 vs 89% in patients with an Agatston score <1000). This implies that significant coronary calcification still yields interpretive challenges. Soschynski et al^154^ demonstrated in scans obtained by photon-counting CT scanners, a significant decrease in accessibility of segments in patients with a calcium score >600, worsening at Agatston scores >900.

With positive results in studies showing high accuracy of the detection of CAD, in certain high-risk patients, UHR photon counting CT shows promise to overcome certain limitations of conventional CT. However, further validation in larger groups is necessary to verify which patients benefit most from this new CT scan, given the decreased accessibility in patients with high calcium scores.

## Summary and future directions

Coronary computed tomography angiography has developed into a reliable, noninvasive imaging technique for the evaluation of patients with suspected atherosclerosis, with high accuracy for the detection and exclusion of CAD. Cardiovascular risk increases gradually as stenosis severity or plaque extent increases, and CCTA improves risk prediction throughout the entirety of the disease burden. Advances in CT technologies allow detailed atherosclerotic assessment of the whole coronary tree including atherosclerosis quantification and assessment of PCAT around the coronary arteries. Plaque location, morphology, burden, and diameter stenosis all affect cardiovascular risk and have been combined in different risk scores. Quantification of low attenuation plaque, a sign of HRP with a high likelihood of developing ACS has shown to be important in risk assessment. The dense calcified plaque on the other side of the HU spectrum presents the end stage of the atherosclerotic plaque and appears to denote most stable plaques with a lower incidence of ACS. However, currently, there are no clinically useful thresholds available to assist with understanding a patient's disease extent and to provide guidance for diagnosis and management, in contrast with CAC scoring and an overall assessment of plaque burden.

Changes in plaque over time can be detected by serial CCTA. Although a worse prognosis is linked to plaque progression, it is not yet known if focusing on the total amount of plaque is the best target to guide clinical management of the patient. With expansion of available therapies to halt progression and events, finding the “vulnerable patient” seems most important. CCTA used as a screening tool in high-risk, asymptomatic patients, might potentially be the future, given that many MI arise from asymptomatic lesions and that CACS solely identifies calcified plaque. However, additional studies are warranted to evaluate the incremental role of CCTA over CACS in this scenario. New advances in CT technology, such as photon counting CT have shown promising results and may be particularly helpful with visualization of intraluminal stents, smaller coronary segments, and lesions with heavy calcification. The CCTA evidence has amassed rapidly over the past decade and newer approaches and guided therapy based on plaque findings will certainly be a strong part of future research findings.
